# Supplementary material for: Strengthening TB care pathways: Integrating informal and AYUSH practitioners for early diagnosis and referral
Source: PLOS Glob Public Health. 2026 Apr 8;6(4):e0004416. doi: 10.1371/journal.pgph.0004416 (PMC13061243; doi:10.1371/journal.pgph.0004416)
Supplement: S1 Appendix — The text also includes additional tables to support understanding relationship of comorbidities, symptom severity and occupation on patient pathway (choice of first point of care, first provider type, and mean values of patient delay & diagnostic delay). (DOCX) [file pgph.0004416.s001.docx]

**Strengthening TB Care Pathways: Integrating Informal and AYUSH Practitioners for Early Diagnosis and Referral**

**Authors**: Ridhima Sodhi^1*^, Shruti Goel^1^, Rohit Baghel^1^, Kiran Rade^2 3^, Kshitij Khaparde^2^, Harkesh Dabas^1^, Shamim Mannan^1^, Manoj Singh^1^

1. William J Clinton Foundation, New Delhi, India
2. World Health Organization, Country Office for India
3. Stop TB Partnership, United Nations Office for Project Services (UNOPS), Geneva, Switzerland

**S1 Appendix**

This section provides additional analytical analyses to support the thematic discussion points presented in the paper. While these findings have been integrated into the broader narrative of the study, they may not be explicitly detailed throughout the manuscript. These analyses are intended to enrich the reader's understanding and reinforce key insights discussed in the paper.

## Regression Modeling Equation

The standard modeling equation used is as below:

$$Y_{it}=\alpha+ \beta_{1}{Intervention}_{i}+ \beta_{2} \left( {Intervention}_{i}*{Time}_{t} \right)+\beta_{3}{Time}_{t}\boldsymbol{+}X_{it}+ \varepsilon_{it}$$

where,

- $Y_{it}$​ is the outcome variable for unit i at time t.
- ${Intervention}_{i}$​ is a binary indicator equal to 1 if the unit is in the treatment group and 0 if in the control group.
- ${Time}_{t}$ is a binary indicator equal to 1 if the time-period is after the intervention (endline) and 0 if before the intervention (baseline).
- ${Intervention}_{i}*{Time}_{t}$​ is the interaction term representing the difference-in-differences estimator.
- Covariates $X_{it}$​ include age, gender, occupation, comorbidity, symptom severity, first point of contact, and other relevant individual and provider-level characteristics
- α is the intercept.
- $\beta_{1}$ captures the difference between treatment and control groups before the intervention. Since we did not have an intervention group in the baseline, this coefficient is null for our model.
- $\beta_{2}$ is the DiD estimator, which represents the treatment effect. In other words, it measures the additional effect of the intervention on the treatment group, over and above any time trends
- $\beta_{3}$​ captures the common time trend affecting both groups. In our case, it affects the evolution of healthcare systems through the baseline and endline period.
- $\varepsilon_{it}$​ is the error term.

## Multicollinearity for OLS estimation for Patient Delay

**Table A. Multicollinearity diagnostics for the OLS model of patient delay (n = 304)**
*Adjusted Generalized Variance Inflation Factors (GVIF) values; GVIF^(1/(2×Df)).*

All adjusted values were well below the commonly accepted threshold of 2, indicating no evidence of problematic multicollinearity among included covariates

|  | **GVIF** | **Df** | **Adjusted GVIF** |
| --- | --- | --- | --- |
| **intervention** | 1.68 | 1.00 | 1.30 |
| **time** | 3.05 | 1.00 | 1.75 |
| **age** | 1.29 | 1.00 | 1.14 |
| **gender** | 1.38 | 1.00 | 1.18 |
| **occupation** | 1.98 | 4.00 | 1.09 |
| **type.hh.joint** | 1.15 | 1.00 | 1.07 |
| **first.poc** | 1.39 | 3.00 | 1.06 |
| **comorb.binary** | 1.43 | 1.00 | 1.20 |
| **tb.history.yes.no** | 1.07 | 1.00 | 1.04 |
| **symptom.severity** | 1.14 | 1.00 | 1.07 |
| **prov.choosing.reason** | 2.09 | 4.00 | 1.10 |
| **sym.n** | 1.96 | 1.00 | 1.40 |

## Multicollinearity for OLS estimation for Diagnostic Delay

**Table B. Multicollinearity diagnostics for the OLS model of diagnostic delay (n = 304)**
*Adjusted Generalized Variance Inflation Factors (GVIF) values; GVIF^(1/(2×Df)).*

All adjusted values were well below the commonly accepted threshold of 2, indicating no evidence of problematic multicollinearity among included covariates

|  | **GVIF** | **Df** | **Adjusted GVIF** |
| --- | --- | --- | --- |
| **intervention** | 1.63 | 1.00 | 1.28 |
| **time** | 1.78 | 1.00 | 1.34 |
| **age** | 1.21 | 1.00 | 1.10 |
| **gender** | 1.04 | 1.00 | 1.02 |
| **type.hh.joint** | 1.05 | 1.00 | 1.02 |
| **first.doc.type** | 1.26 | 3.00 | 1.04 |
| **comorb.binary** | 1.23 | 1.00 | 1.11 |
| **symptom.severity** | 1.07 | 1.00 | 1.03 |
| **tb.history.yes.no** | 1.02 | 1.00 | 1.01 |

## Multicollinearity for OLS estimation for N - HCPs

**Table C. Multicollinearity Diagnostics for the Regression Model of Number of Providers Visited (N-HCP)**

*Adjusted Generalized Variance Inflation Factors (GVIF) values; GVIF^(1/(2×Df)).*

|  | **GVIF** | **Df** | **Adjusted GVIF** |
| --- | --- | --- | --- |
| **intervention** | 1.66 | 1.00 | 1.29 |
| **time** | 2.19 | 1.00 | 1.48 |
| **age** | 1.27 | 1.00 | 1.13 |
| **gender** | 1.33 | 1.00 | 1.16 |
| **type.hh.joint** | 1.12 | 1.00 | 1.06 |
| **occupation** | 1.87 | 4.00 | 1.08 |
| **symptom.severity** | 1.14 | 1.00 | 1.07 |
| **first.doc.type** | 1.56 | 3.00 | 1.08 |
| **comorb.binary** | 1.42 | 1.00 | 1.19 |
| **tb.history.yes.no** | 1.07 | 1.00 | 1.04 |
| **prov.choosing.reason** | 2.31 | 4.00 | 1.11 |

## Patient Delay – QTE PLOT

**Fig A**

*Quantile Treatment Effect (QTE) of the intervention on patient delay.*

The plot shows the estimated effect of the intervention across quantiles (τ), with 95% confidence intervals. No statistically significant effect was observed at any point in the distribution, consistent with the null findings from the OLS and quantile regression models


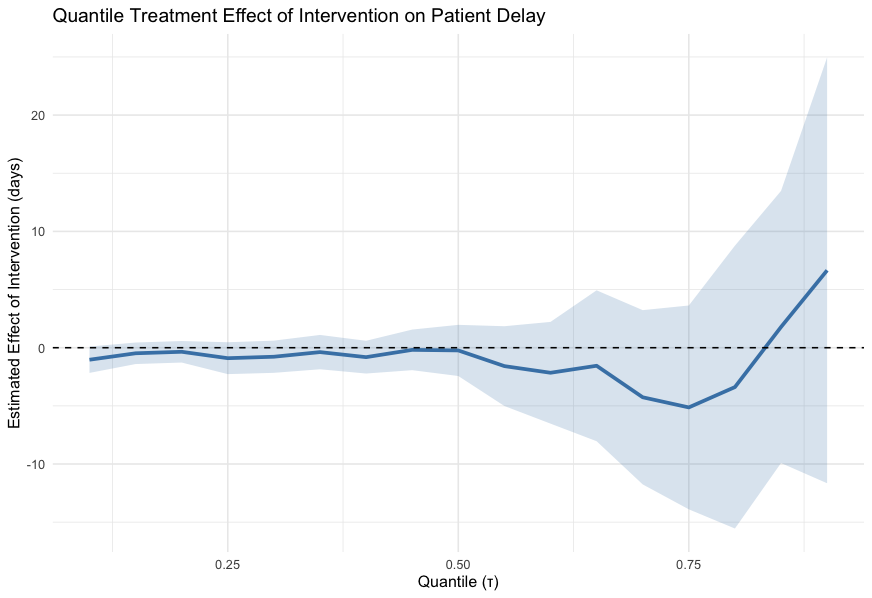


## Diagnostic Delay – QTE PLOT

The Quantile Treatment Effect (QTE) analysis for diagnostic delay revealed no meaningful variation in the intervention effect across quantiles, with estimates effectively tracing a flat line at zero. Given the absence of visual differentiation, the corresponding plot is not presented.

## Diagnostic Delay – Supplementary Regression Analysis

Model Specification:

lm.1<-lm(diagnostic.delay ~ intervention*first.doc.type + time + age + gender + type.hh.joint + n.docs.visited + comorb.binary + symptom.severity+ tb.history.yes.no , data=demo.inter)

**Table D. OLS Model with Diagnostic Delay as Dependent Variable; Interaction Term on Type of First Provider**

|  | OLS (Robust SE) |
| --- | --- |
| intervention | -8.86** (-16.88, -0.85) |
| first.doc.typeayush | 18.19 (-40.32, 76.69) |
| first.doc.typeself/pharmacy | -1.26 (-9.28, 6.75) |
| first.doc.typeunqualified | 7.46 (-7.95, 22.87) |
| time | -10.64 (-24.99, 3.70) |
| age | 0.07 (-0.19, 0.32) |
| gendermale | -1.54 (-8.17, 5.10) |
| type.hh.joint | 1.27 (-5.57, 8.11) |
| n.docs.visited | 2.13 (-2.82, 7.09) |
| comorb.binary | 6.22* (-0.79, 13.23) |
| symptom.severitysevere | 1.73 (-5.10, 8.56) |
| tb.history.yes.no | -2.33 (-10.16, 5.50) |
| intervention:first.doc.typeayush | -10.73 (-69.02, 47.56) |
| intervention:first.doc.typeself/pharmacy | 24.62 (-7.63, 56.88) |
| intervention:first.doc.typeunqualified | -8.39 (-24.99, 8.21) |
| Constant | 18.22* (-0.40, 36.84) |
| Observations | 304 |
| R^2^ | 0.14 |
| Adjusted R^2^ | 0.1 |
| Residual Std. Error | 31.05 (df = 288) |
| F Statistic | 3.16^***^ (df = 15; 288) |
| *Note:*p<0.05; **p<0.01; ***p<0.001* | |

## Number of Providers Visited– Supplementary Regression Analysis

Model Specification:

poisson_model.2 <- glm(n.docs.visited ~ intervention*first.doc.type + time + age + gender + type.hh.joint + occupation+ symptom.severity+ comorb.binary + tb.history.yes.no + prov.choosing.reason, family = poisson(link = "log"), data = demo.inter)

**Table E. Poisson Model with number of providers as dependent variable; Interaction Term on Type of First Provider**

|  | Risk Ratios (Robust Confidence Intervals) |
| --- | --- |
| intervention | 0.89*** (0.59, 1.19) |
| time | 0.51*** (0.23, 0.79) |
| first.doc.typeayush | 1.48*** (0.79, 2.18) |
| first.doc.typeself/pharmacy | 1.30*** (1.00, 1.60) |
| first.doc.typeunqualified | 1.34*** (1.08, 1.59) |
| age | 1.00 (0.99, 1.01) |
| gendermale | 0.96 (0.76, 1.16) |
| type.hh.joint | 0.94** (0.76, 1.11) |
| occupationgovt | 0.97 (0.49, 1.46) |
| occupationprivate | 1.04 (0.76, 1.31) |
| occupationretired/unemp | 0.93* (0.71, 1.15) |
| occupationself employed | 1.00 (0.56, 1.43) |
| symptom.severitysevere | 0.91*** (0.72, 1.10) |
| comorb.binary | 1.02 (0.81, 1.23) |
| tb.history.yes.no | 0.93* (0.71, 1.15) |
| prov.choosing.reasonknown/family networks | 0.93 (0.62, 1.25) |
| prov.choosing.reasonothers | 0.96 (0.51, 1.41) |
| prov.choosing.reasonproximity | 0.92 (0.64, 1.20) |
| prov.choosing.reasonreferral | 1.00 (0.67, 1.32) |
| intervention:first.doc.typeayush | 1.29** (0.48, 2.10) |
| intervention:first.doc.typeself/pharmacy | 1.42*** (0.91, 1.93) |
| intervention:first.doc.typeunqualified | 1.49*** (0.98, 2.00) |
| Constant | 2.75*** (2.34, 3.15) |
| Observations | 304 |
| Akaike Inf. Crit. | 823.36 |
| *Note:*p<0.1; **p<0.05; ***p<0.01;  1. There was 1 retired individual, who was clubbed with unemployed* | |

## Understanding relationship of comorbidities on patient pathway

**Table F**

*Understanding the choice of first point of care, first provider type, and mean values of patient delay & diagnostic delay, segregated by comorbidities*

|  |  | **Status of Comorbidities** | |  |
| --- | --- | --- | --- | --- |
|  |  | **0**, N = 43*^1^* | **1**, N = 58*^1^* | **p-value***^2^* |
| Baseline | **First POC** |  |  | 0.6 |
|  | ayush | 1 (2.3%) | 1 (1.7%) |  |
|  | chw | 1 (2.3%) | 5 (8.6%) |  |
|  | friends/family | 40 (93%) | 45 (78%) |  |
|  | pharmacy | 0 (0%) | 3 (5.2%) |  |
|  | unqualified | 1 (2.3%) | 4 (6.9%) |  |
|  | **First Doc** |  |  | 0.7 |
|  | allopathic | 21 (49%) | 26 (45%) |  |
|  | ayush | 0 (0%) | 1 (1.7%) |  |
|  | self/pharmacy | 6 (14%) | 17 (29%) |  |
|  | unqualified | 16 (37%) | 14 (24%) |  |
|  | **Patient Delay (PD)** | |  | 0.3 |
|  | Median (IQR) | 33 (30, 47) | 40 (30, 55) |  |
|  | Mean | 37 | 45 |  |
|  | **Diagnostic Delay (DxD)** | |  | 0.005 |
|  | Median (IQR) | 23 (16, 30) | 28 (22, 36) |  |
|  | Mean | 25 | 37 |  |
|  |  | **0**, N = 53*^1^* | **1**, N = 27*^1^* | **p-value***^2^* |
| Endline Control | **First POC** |  |  | 0.069 |
|  | ayush | 1 (1.9%) | 0 (0%) |  |
|  | chw | 2 (3.8%) | 0 (0%) |  |
|  | friends/family | 40 (75%) | 18 (67%) |  |
|  | unqualified | 2 (3.8%) | 0 (0%) |  |
|  | qualified | 8 (15%) | 9 (33%) |  |
|  | **First Doc** |  |  | >0.9 |
|  | allopathic | 41 (77%) | 21 (78%) |  |
|  | ayush | 2 (3.8%) | 1 (3.7%) |  |
|  | self/pharmacy | 1 (1.9%) | 0 (0%) |  |
|  | unqualified | 9 (17%) | 5 (19%) |  |
|  | **Patient Delay (PD)** | |  | 0.005 |
|  | Median (IQR) | 9 (7, 16) | 15 (10, 31) |  |
|  | Mean | 14 | 25 |  |
|  | **Diagnostic Delay (DxD)** | |  | 0.9 |
|  | Median (IQR) | 3 (1, 10) | 4 (1, 23) |  |
|  | Mean | 15 | 21 |  |
|  |  | **0**, N = 90*^1^* | **1**, N = 33*^1^* | **p-value***^2^* |
| Endline Intervention | **First POC** |  |  | 0.4 |
|  | ayush | 3 (3.3%) | 0 (0%) |  |
|  | chw | 0 (0%) | 1 (3.0%) |  |
|  | friends/family | 73 (81%) | 29 (88%) |  |
|  | pharmacy | 1 (1.1%) | 0 (0%) |  |
|  | unqualified | 3 (3.3%) | 1 (3.0%) |  |
|  | qualified | 10 (11%) | 2 (6.1%) |  |
|  | **First Doc** |  |  | 0.4 |
|  | allopathic | 57 (63%) | 20 (61%) |  |
|  | ayush | 16 (18%) | 0 (0%) |  |
|  | self/pharmacy | 9 (10%) | 8 (24%) |  |
|  | unqualified | 8 (8.9%) | 5 (15%) |  |
|  | **Patient Delay (PD)** | |  | 0.4 |
|  | Median (IQR) | 9 (7, 15) | 13 (7, 21) |  |
|  | Mean | 18 | 16 |  |
|  | **Diagnostic Delay (DxD)** | |  | 0.1 |
|  | Median (IQR) | 4 (1, 8) | 2 (0, 5) |  |
|  | Mean | 9 | 13 |  |

## Understanding relationship of symptom severity on patient pathway

**Table G**

*Understanding the choice of first point of care, first provider type, and mean values of patient delay & diagnostic delay, segregated by symptom severity*

|  |  | **Symptom Severity** | |  |
| --- | --- | --- | --- | --- |
|  |  | **mild/moderate**, N = 75*^1^* | **severe**, N = 26*^1^* | **p-value***^2^* |
| Baseline | **First PoC** |  |  | 0.5 |
|  | ayush | 1 (1.3%) | 1 (3.8%) |  |
|  | chw | 6 (8.0%) | 0 (0%) |  |
|  | friends/family | 60 (80%) | 25 (96%) |  |
|  | pharmacy | 3 (4.0%) | 0 (0%) |  |
|  | unqualified | 5 (6.7%) | 0 (0%) |  |
|  | **First Doc** |  |  | 0.15 |
|  | allopathic | 32 (43%) | 15 (58%) |  |
|  | ayush | 1 (1.3%) | 0 (0%) |  |
|  | self/pharmacy | 17 (23%) | 6 (23%) |  |
|  | unqualified | 25 (33%) | 5 (19%) |  |
|  | **Patient Delay (PD)** | |  | 0.029 |
|  | Median (IQR) | 40 (30, 55) | 30 (27, 40) |  |
|  | Mean | 44 | 34 |  |
|  | **Diagnostic Delay (DxD)** | |  | 0.075 |
|  | Median (IQR) | 28 (22, 35) | 24 (14, 32) |  |
|  | Mean | 34 | 25 |  |
|  |  | **mild/moderate**, N = 46*^1^* | **severe**, N = 34*^1^* | **p-value***^2^* |
| Endline Control | **First PoC** |  |  | 0.6 |
|  | ayush | 0 (0%) | 1 (2.9%) |  |
|  | chw | 2 (4.3%) | 0 (0%) |  |
|  | friends/family | 32 (70%) | 26 (76%) |  |
|  | unqualified | 1 (2.2%) | 1 (2.9%) |  |
|  | qualified | 11 (24%) | 6 (18%) |  |
|  | **First Doc** |  |  | 0.5 |
|  | allopathic | 37 (80%) | 25 (74%) |  |
|  | ayush | 1 (2.2%) | 2 (5.9%) |  |
|  | self/pharmacy | 0 (0%) | 1 (2.9%) |  |
|  | unqualified | 8 (17%) | 6 (18%) |  |
|  | **Patient Delay (PD)** | |  | 0.6 |
|  | Median (IQR) | 11 (7, 22) | 10 (7, 17) |  |
|  | Mean | 18 | 17 |  |
|  | **Diagnostic Delay (DxD)** | |  | 0.4 |
|  | Median (IQR) | 4 (0, 12) | 4 (1, 15) |  |
|  | Mean | 18 | 15 |  |
|  |  | **mild/moderate**, N = 67*^1^* | **severe**, N = 56*^1^* | p-value*^2^* |
| Endline Intervention | **First POC** |  |  | 0.4 |
|  | ayush | 1 (1.5%) | 2 (3.6%) |  |
|  | chw | 0 (0%) | 1 (1.8%) |  |
|  | friends/family | 56 (84%) | 46 (82%) |  |
|  | pharmacy | 1 (1.5%) | 0 (0%) |  |
|  | unqualified | 1 (1.5%) | 3 (5.4%) |  |
|  | qualified | 8 (12%) | 4 (7.1%) |  |
|  | **First Doc** |  |  | 0.13 |
|  | allopathic | 46 (69%) | 31 (55%) |  |
|  | ayush | 8 (12%) | 8 (14%) |  |
|  | self/pharmacy | 7 (10%) | 10 (18%) |  |
|  | unqualified | 6 (9.0%) | 7 (13%) |  |
|  | **Patient Delay (PD)** | |  | 0.2 |
|  | Median (IQR) | 10 (7, 17) | 8 (6, 15) |  |
|  | Mean | 20 | 15 |  |
|  | **Diagnostic Delay (DxD)** | |  | 0.029 |
|  | Median (IQR) | 2 (1, 6) | 5 (2, 11) |  |
|  | Mean | 5 | 17 |  |

## Understanding relationship of occupation on patient pathway

**Table H**

*Understanding the choice of first point of care, first provider type, and mean values of patient delay & diagnostic delay, segregated by occupation of the individual*

|  |  | **agri/labor,**  **N = 461** | **govt,**  **N = 31** | **private,**  **N = 121** | **self.emp,**  **N = 0** | **unemp,**  **N = 401** | **retired**,  N = 0 | **p-value***^2^* |  | |
| --- | --- | --- | --- | --- | --- | --- | --- | --- | --- | --- |
| Baseline | **First POC** |  |  |  |  |  |  | 0.5 |  | |
|  | ayush | 1 (2.2%) | 0 (0%) | 0 (0%) |  | 1 (2.5%) |  |  |  | |
|  | chw | 2 (4.3%) | 0 (0%) | 2 (17%) |  | 2 (5.0%) |  |  |  | |
|  | friends/family | 37 (80%) | 3 (100%) | 9 (75%) |  | 36 (90%) |  |  |  | |
|  | pharmacy | 2 (4.3%) | 0 (0%) | 1 (8.3%) |  | 0 (0%) |  |  |  | |
|  | unqualified | 4 (8.7%) | 0 (0%) | 0 (0%) |  | 1 (2.5%) |  |  |  | |
|  | **First Doc** |  |  |  |  |  |  | 0.2 |  | |
|  | allopathic | 18 (39%) | 2 (67%) | 8 (67%) |  | 19 (48%) |  |  |  | |
|  | ayush | 1 (2.2%) | 0 (0%) | 0 (0%) |  | 0 (0%) |  |  |  | |
|  | self/pharmacy | 11 (24%) | 1 (33%) | 3 (25%) |  | 8 (20%) |  |  |  | |
|  | unqualified | 16 (35%) | 0 (0%) | 1 (8.3%) |  | 13 (33%) |  |  |  | |
|  | **Patient Delay (PD)** | |  |  |  |  |  | 0.057 |  | |
|  | Median (IQR) | 40 (31, 55) | 30 (28, 30) | 30 (26, 34) |  | 38 (30, 46) |  |  |  | |
|  | Mean | 47 | 29 | 34 |  | 39 |  |  |  | |
|  | **Diagnostic Delay (DxD)** | |  |  |  |  |  | 0.7 |  | |
|  | Median (IQR) | 25 (20, 31) | 29 (26, 31) | 28 (21, 34) |  | 28 (22, 42) |  |  |  | |
|  | Mean | 29 | 28 | 28 |  | 37 |  |  |  | |
|  |  | **agri/labor, N = 221** | **govt, N = 41** | **private, N = 91** | **self.emp, N = 81** | **unemp, N = 371** | **retired**, N = 0 | **p-value***^2^* |  | |
| Endline Control | **First POC** |  |  |  |  |  |  | 0.064 |  | |
|  | ayush | 1 (4.5%) | 0 (0%) | 0 (0%) | 0 (0%) | 0 (0%) |  |  |  | |
|  | chw | 1 (4.5%) | 0 (0%) | 0 (0%) | 1 (13%) | 0 (0%) |  |  |  | |
|  | friends/family | 17 (77%) | 2 (50%) | 6 (67%) | 7 (88%) | 26 (70%) |  |  |  | |
|  | unqualified | 0 (0%) | 0 (0%) | 0 (0%) | 0 (0%) | 2 (5.4%) |  |  |  | |
|  | allopathic | 3 (14%) | 2 (50%) | 3 (33%) | 0 (0%) | 9 (24%) |  |  |  | |
|  | **First Doc** |  |  |  |  |  |  | 0.2 |  | |
|  | allopathic | 14 (64%) | 4 (100%) | 9 (100%) | 6 (75%) | 29 (78%) |  |  |  | |
|  | ayush | 2 (9.1%) | 0 (0%) | 0 (0%) | 0 (0%) | 1 (2.7%) |  |  |  | |
|  | self/pharmacy | 0 (0%) | 0 (0%) | 0 (0%) | 0 (0%) | 1 (2.7%) |  |  |  | |
|  | unqualified | 6 (27%) | 0 (0%) | 0 (0%) | 2 (25%) | 6 (16%) |  |  |  | |
|  | **Patient Delay (PD)** | |  |  |  |  |  | 0.6 |  | |
|  | Median (IQR) | 10 (7, 16) | 17 (14, 19) | 10 (5, 25) | 11 (9, 51) | 10 (8, 17) |  |  |  | |
|  | Mean | 17 | 17 | 17 | 32 | 15 |  |  |  | |
|  | **Diagnostic Delay (DxD)** | |  |  |  |  |  | 0.14 |  | |
|  | Median (IQR) | 4 (1, 8) | 20 (6, 67) | 6 (1, 16) | 0 (0, 5) | 3 (1, 16) |  |  |  | |
|  | Mean | 18 | 53 | 14 | 4 | 16 |  |  |  | |
|  |  | **agri/labor**, N = 27*^1^* | **govt**, N = 5*^1^* | **private**, N = 22*^1^* | **self.emp**, N = 10*^1^* | **unemp**, N = 58*^1^* | **retired**, N = 1*^1^* | **p-value***^2^* |  | |
| Endline Intervention | **First POC** |  |  |  |  |  |  | 0.4 |  | |
|  | ayush | 2 (7.4%) | 0 (0%) | 0 (0%) | 0 (0%) | 1 (1.7%) | 0 (0%) |  |  | |
|  | chw | 1 (3.7%) | 0 (0%) | 0 (0%) | 0 (0%) | 0 (0%) | 0 (0%) |  |  | |
|  | friends/family | 22 (81%) | 4 (80%) | 18 (82%) | 8 (80%) | 49 (84%) | 1 (100%) |  |  | |
|  | pharmacy | 0 (0%) | 0 (0%) | 0 (0%) | 1 (10%) | 0 (0%) | 0 (0%) |  |  | |
|  | unqualified | 1 (3.7%) | 0 (0%) | 0 (0%) | 1 (10%) | 2 (3.4%) | 0 (0%) |  |  | |
|  | allopathic | 1 (3.7%) | 1 (20%) | 4 (18%) | 0 (0%) | 6 (10%) | 0 (0%) |  |  | |
|  | **First Doc** |  |  |  |  |  |  | 0.5 |  | |
|  | allopathic | 15 (56%) | 3 (60%) | 17 (77%) | 6 (60%) | 36 (62%) | 0 (0%) |  |  | |
|  | ayush | 3 (11%) | 0 (0%) | 1 (4.5%) | 1 (10%) | 11 (19%) | 0 (0%) |  |  | |
|  | self/pharmacy | 5 (19%) | 2 (40%) | 3 (14%) | 1 (10%) | 5 (8.6%) | 1 (100%) |  |  | |
|  | unqualified | 4 (15%) | 0 (0%) | 1 (4.5%) | 2 (20%) | 6 (10%) | 0 (0%) |  |  | |
|  | **Patient Delay (PD)** | |  |  |  |  |  | 0.3 |  | |
|  | Median (IQR) | 8 (6, 10) | 11 (7, 16) | 10 (6, 28) | 15 (12, 29) | 10 (7, 18) | 7 (7, 7) |  |  | |
|  | Mean | 10 | 15 | 26 | 19 | 18 | 7 |  |  | |
|  | **Diagnostic Delay (DxD)** | |  |  |  |  |  | 0.2 |  | |
|  | Median (IQR) | 5 (3, 10) | 1 (0, 5) | 2 (1, 6) | 5 (0, 11) | 3 (1, 6) | 18 (18, 18) |  |  | |
|  | Mean | 16 | 3 | 5 | 29 | 7 | 18 |  |  | |
|  | *^1^* n (%) | | | | | | | | |  |
|  | *^2^* Kruskal-Wallis rank sum test | | | | | | | | |  |
