# Supplementary material for: Strengthening TB care pathways: Integrating informal and AYUSH practitioners for early diagnosis and referral
Source: PLOS Glob Public Health. 2026 Apr 8;6(4):e0004416. doi: 10.1371/journal.pgph.0004416 (PMC13061243; doi:10.1371/journal.pgph.0004416)
Supplement: Attachment — Submitted filename: Response to Reviewers.docx [file pgph.0004416.s003.docx]

**Note of thanks**

We sincerely thank the editor and reviewer for reading through our manuscript, and giving their valuable feedback.

In particular, we are grateful to the editor for highlighting areas which can benefit from improvement and clarity, and hence – be more useful for the audience at large. The constructive feedback provided by your office has helped us in enhancing the clarity, quality, and rigor of our manuscript, and the same are reflected in the revised manuscript.

We have also responded to specific queries here, and will be looking forward to the journal’s response.

*Authors’ responses to editor’s and reviewer’s comments are mentioned in green*

# Funding Statement (now edited)

1. We have amended your Competing Interest statement to comply with journal style. We kindly ask that you double check the statement and let us know if anything is incorrect.

 Thank you. We checked, and the same is fine.

2. Your current Financial Disclosure states, “The funding for both the baseline and endline studies was provided by Wave 08 of the TB REACH Program, granted to the William J. Clinton Foundation. The authors did not receive any funding for data compilation, subsequent analysis, or manuscript preparation.”. However, your funding information on the submission form indicates that you did not receive funding. Please indicate by return email the full and correct funding information for your study and confirm the order in which funding contributions should appear. Please be sure to indicate whether the funders played any role in the study design, data collection and analysis, decision to publish, or preparation of the manuscript.

Thank you for this observation. We would like to clarify that while the baseline and endline assessments were conducted under a project funded by Wave 08 of the TB REACH program (Stop TB Partnership), the project deliverables for the same were operational reports – and not manuscript writing.

In other words, the authors did not receive any additional funding for data compilation, statistical analysis, or manuscript preparation, and all of the related work undertaken falls outside the scope of the original project funding.

We also confirm that the funders (Stop TB Partnership / TB REACH) had no role in the study design, data collection and analysis, decision to publish, or preparation of the manuscript.

We have stated the below statement in the revised submission.

# *Funding: The baseline and endline assessments were funded by Wave 08 of the TB REACH program (Stop TB Partnership), awarded to the William J. Clinton Foundation. This grant supported the research and report preparation – but did not cover manuscript preparation.*

# *The Stop TB partnership had no involvement in study design, data collection, analysis, the decision to publish, or manuscript preparation. The authors received no additional funding for data compilation, statistical analysis, or manuscript preparation.*

# Additional Editor Comments (if provided):

**author responses in green*

This manuscript presents a timely and methodologically interesting quasi-experimental mixed-methods study assessing the integration of informal and AYUSH providers in the TB care pathway. However, several methodological and procedural concerns need to be addressed to strengthen the manuscript's scientific rigor and clarity.

Thank You for your suggestions. We have edited the manuscript after going through your feedback, and have also answered specific queries in this document.

We believe the revised version addresses your concerns, and we look forward to additional feedback.

## 1. Selection and Definition of Providers

- The manuscript lacks a clear operational definition of "quacks" (unqualified providers). Also using the term “quacks” - while it is colloquially understood, it is pejorative, stigmatizing, and unprofessional in a scholarly context. How were they distinguished from AYUSH certified providers or informal allopathic practitioners? Were any formal criteria used for this classification (e.g., registration status, community nomination, verification by local health authorities)?

Thank you for this – please find our answers below:

1. **Using the “quack” term.**

Thanks for raising this question – it has made us re-consider about the decision to use this term. Firstly, we would like to share that we agree with your suggestion, and have replaced the term ‘quack’ with “unqualified provider” in the revised manuscript.

Our manuscript centres on the potential contributions of an integrated healthcare system, which includes all different kinds of individuals and medical professionals – relied upon by the public at large. We, hence, would not like to use any pejorative or stigmatizing term.

Having said that, we do want to share that we had kept the term “**quack**” to ensure authenticity – and were unintentionally ignorant of its stigmatizing context. There exist several analysis in internationally peer-reviewed literature, wherein the term has been used – which we had relied on for reference.

2. **Criterion for classification**

Operationally, individuals who have not been medically trained were referred to as “quacks” in our manuscript. This is separate from those who have been trained in western or allopathic medical training (MBBS / MD), or/and alternative medical practitioners which are AYUSH certified (Ayurveda, Yoga, Naturopathy, Unani, Siddha, and Homeopathy).

The operational criterion used was the “registration status” of the provider.

- **Formal providers** included those who had an allopathic medicine degree (at least an MBBS), and an ongoing practice
- **AYUSH** providers included those with an AYUSH degree and practice – these formally include Ayurvedic, Yogic & Naturopathy, Unani, Siddha, and Homeopathy practitioners. In our project exercise, we did not include “Yogic” and “Naturopathy” practitioners.
  - Ayurvedic practitioner requires Bachelor of Ayurvedic Medicine and Surgery, **BAMS**;
  - Unani practitioner requires Bachelor of Unani Medicine and Surgery or **B.U.M.S**; and,
  - Homeopathic practitioner requires a Bachelor of Homeopathic Medicine and Surgery or **BHMS**).
- **Quacks (now changed to informal providers)** were those for whom no medical registration is available, and hence – are posed to be individuals without any medical degree
- **What was the total provider universe, and what proportion of it was engaged in the project? The representativeness and potential selection bias in provider engagement are unclear.**

Thanks – we are happy to provide these details.

A total of ~**1660 providers were mapped** through the project duration. Of these, **64% or 1069 providers agreed to be engaged** with the project. This engagement is confirmed through either the provider’s attendance in CME (continued medical education) events organized by the project team, a verbal agreement to notify TB patients with the support of our field staff, or/and a memorandum of understanding (MoU).

Just an agreement to engagement does not mean the provider was “actively engaged”. This active engagement is confirmed if the provider allows the field staff to support notification of patients on the government data management system for TB (Nikshay), or themselves conduct that activity. Even a patient referral – for further testing, or to another (qualified) medical practitioner, will count as actual activity.

In sum, a provider was “**actively engaged**” if they refer at least one presumptive TB patient for further diagnostic testing or to another provider.

This accounted for **35% of mapped, and 54% of those who engaged** – across the provider universe, totaling **582 unique providers**.

## 2. Provider Selection and Mapping Process:

- The selection process for engaging informal and AYUSH providers is not well explained. Was snowball sampling, community health worker mapping, or an NGO supported network used?

For the most part, provider engagement was hugely dependent on their own willingness – and we attempted to include all different providers in Durg, Chhattisgarh, the area of the project.

AYUSH and chemists / pharmacies contribute to ~40% of presumptive TB cases in the area – and we took support from our operational staff, as well as the District TB Officer’s (DTO’s) office, in mapping all of the providers and pharmacies in the area. The only ones not initiated for engagement were those who, as per the DTO office, had negligible footfall of patients (in case of AYUSH clinics), or those who did not dispense TB medications (in case of pharmacies).

- Were there any providers who declined to participate? What were the reasons?

Yes, as also evident in the engagement vs active provider numbers – multiple providers declined to participate. While specific reasons were not captured for each and every provider, the typical reasons to not engage is a) thinking of it as a time commitment, and b) unwillingness in sharing patient data. It is also safe to say that the engagement incentives were not enough for them to engage.

In our project, we also required the providers to send the presumptive TB patients to empanelled CxR labs for screening – which may also have been an impediment – as many providers have pre-existing ties with diagnostic labs.

- Were any systematic exclusion criteria applied to avoid engaging potentially unethical or unreliable providers?

There was no systematic criteria – we typically relied on the combination of our mapping activity and DTO’s office for guidance in this matter.

Since we attempted to engage all different providers, and more importantly - our incentives were linked to an actual TB diagnosis, we did not record any evidence of a provider who is unethical or unreliable from that context.

## 3. Training and Standardization:

- What standardized training, if any, was provided to informal and AYUSH providers? How was their competency in identifying and referring presumptive TB patients ensured?

**Training:**

Standardized training and CMEs were organized for all providers – encompassing allopathic practitioners, AYUSH, pharmacies, and unqualified. The training included trainings / education sessions covering awareness about the TB disease, common symptoms to look for, and the latest NTEP guidelines on disease management.

Providers were also made aware of the significance of CxR screening and further diagnostic testing through NAATs, as well as the benefits of patient and caregiver counselling.

Through the project – a total of 18 in-person CMEs were organized.

**Competency:**

The project tried to ensure competency through a combination of training and incentives. So while CxR screening was free of cost for all presumptive TB patients, a provider referral would only trigger incentive if they were diagnosed with TB.

- Was provider adherence to referral protocols monitored throughout the intervention period?

Yes, the team at WJCF (William J Clinton Foundation) actively monitored referral protocols. This was not done in an overbearing manner – in order to ensure engagement. Instead, the team relied on the following measures:

- Pre-set schedule of CME (Continuous medical education) sessions – communicated to providers in advance, and encouraging them to attend
- Use of WhatsApp (broadcast messages and groups)to communicate updates on: a) latest updates on TB compliance, b) infographics on project progress and referral protocols, and c) sharing of any individual queries with resolutions for collective knowledge sharing, among other things
- How was quality assurance handled, especially when involving non-certified practitioners?

As share before, the quality assurance was handled through regular CME sessions, as well as by ensuring that incentive is tied to an actual TB diagnosis.

## 4. Incentives and Risk of Misuse:

- How were financial incentives for successful referrals monitored and regulated? Was there any mechanism to prevent gaming of the system, such as unnecessary referrals?

The financial incentive for successful referral was monitored through an MIS system – which included a dedicated provider portal. In the latter, providers could raise monthly invoices for the service reimbursement.

The incentive, as shared before, was tied to the actual diagnosis of a presumptive TB patient – which worked to ensure that unnecessary referrals may not be made. Additional mechanisms to ensure unnecessary referrals included:

- each patient had to be registered through a unique phone number so the same patient couldn’t be registered multiple times
- all the invoices were reviewed by a finance officer along with submitted proof documents like x-ray report, doctor prescription, and Nikshay Id. If there were inconsistencies wherein the picture was blurry, name didn’t match or incorrect proof documents were uploaded against invoices raised, the case was highlighted to the state program lead. The state lead would then validate the proofs by asking the provider to resubmit the correct proofs.

Thus, a 2-stage verification and validation mechanism ensured each incentive is validated and no unnecessary or incomplete referrals are being incentivized.

- Did the researchers track the conversion rate of referrals to confirmed TB diagnoses by type of referring provider (AYUSH, pharmacy, quack)?

This was tracked under standard programmatic protocol.

Private formal providers had the highest competency – with 13% of their presumptive TB patients (who also got tested), being diagnosed with TB. This was followed by informal providers and pharmacies – for whom the number was 8%. Only about 5% of individuals referred for Chest X Ray by Ayush practitioners were diagnosed with TB.

## 5. DiD Assumptions and Limitations:

- A key assumption of the DiD model is parallel trends. The manuscript does not provide evidence of pre-intervention trend similarity between groups.

Thanks for this observation. We acknowledge that we do not present statistical evidence of pre-intervention trend similarity. However, the study design was built around capturing outcomes from the same underlying population across both periods, with both baseline and endline samples drawn from Durg, Chhattisgarh, using consistent sampling strategies. Hence, the assumption should hold true, in principal.

Importantly, no other significant TB interventions were introduced in this region during the study period, reducing the risk of confounding time-varying shocks.

While we recognize that the parallel trends assumption cannot be empirically tested in our case, the contextual uniformity, stable external environment, and shared sampling frame strengthen its plausibility. We have also taken care to transparently report confidence intervals around effect estimates and used a sample size large enough to support credible subgroup analysis.

## 6. Regression Model Specifications:

- The use of linear regression (OLS) for delay variables is problematic due to right-skewed distributions. Consider using GLM with appropriate link functions or quantile regression.

Thank you for this thoughtful observation. We agree that delay variables such as patient delay and diagnostic delay are positively skewed, which limit the assumptions underlying linear regression.

However, we had still used OLS, as it remains the most easily interpretable, is appropriate for the purposes of estimating average treatment effects, and is robust – especially when paired with robust standard errors. We had also, conducted GLM fitting as part of our internal validation, in order to test for directional significance – although that has not been reported for the sake of conciseness.

Changes in main paper text: However, your comment made us re-consider our judgement. We have, now, included the quintile regression at the 50^th^ & 75^th^ percentile. The former is done to complement the interpretation’s effect at the median, relative to the mean which in turn is estimated via OLS. The 75^th^ percentile regression is estimated to estimate the interpretation’s effect for the longer tail of the distribution – or individuals who are particularly pre-disposed to longer delay times. We do want to share that these results remain consistent in direction and statistical significance.

Note (1): In consideration of the addition of the quantile regression, we have now removed the backward/forward selection regression model. This is done to retain simplicity and conciseness of the results presented.

Note (2): Additionally, convergence issues in quantile regression led us to combine sparse categories (fewer than 10 observations) for two variables: *first point of contact* and *occupation*. These recategorizations were applied only in the regression models and are reflected in the “Regression Tables”; they do not affect the summary statistics presented elsewhere. For consistency and comparability, the same grouping was applied to the OLS model to enable side-by-side presentation of results in a single, collective table. **The same has led to very slight changes in the OLS results presented in the earlier manuscript.** For instance, while earlier it was estimated (by OLS) that patient delay reduced by 19.19 days during the baseline and endline time periods; the same estimate is now at 19.36 days.

In light of improving the modeling specification, we have also modified the regression model for estimating the impact of the intervention on number of providers – from OLS to Poisson. This made more sense as the number of healthcare providers is a continuous discrete variable, which is non-negative.

Additions in the supplementary text: We have also, added Quantile treatment effect (QTE) plots, from τ = 0.1 to 0.9, for patient delay (figure S1). The same is not provided for diagnostic delay, as that was presented as a straight line across y axis = 0; also reflecting the null effect as observed in regression models.

o The model includes many covariates relative to the sample size (n=304), raising concerns of overfitting and multicollinearity.

Thank you for this question. It allows us to put into words some of the decisions we were taking during the construction of this manuscript.

While our model has several covariates – they were selected based on prior literature on care-seeking behaviour and TB diagnostic delays. While the most optimized model (selected using backward / forward selection), had just one variable, we stuck to keeping the different covariates to understand their impact on the outcome variables as well. It is essential that we are not trying to optimize for prediction (which would mean optimizing the R-squared), or even for the model’s precision (as it is not a clinical study), but to add to a broader understanding of how steps to integrate different players into a healthcare system can benefit the identification, diagnosis and treatment of TB patients.

*Our approach, as we now read, also aligns with established frameworks such as the Andersen Behavioural Model, and minimizes the risk of overfitting driven by chance associations.*

Having said that, multicollinearity, needs to be minimized, for the very said reasons as well. We have now incorporated the estimates of generalized variance inflation factors in the supplementary text, and added a line to the effect in the main text as well [Tables S1, S2, and S3]. We wish to share that all covariates had had adjusted GVIF values well below accepted thresholds, indicating no concerning multicollinearity in the final model.

o Adjusted R² is moderate, and model parsimony needs better justification.

While the adjusted R² of our OLS models was moderate, this is not unexpected given the complexity and individual variability in health-seeking behaviour. Importantly, our objective was not to maximize predictive power, but to obtain unbiased estimates of the treatment effect after adjusting for theoretically relevant confounders.

To further ensure parsimony and robustness, we tested model performance using quantile regression. We find that findings are consistent, supporting the reliability of the primary specification.

## 7. Sample and Response Bias:

- Non-response rate was high (~70%), and the potential for selection bias is significant. Was any weighting or adjustment done to account for this?

We acknowledge the high non-response rate (~70%). However, we employed stratified random sampling based on age, gender, and provider type to maintain the representativeness of the underlying population. This is reflected in Table 1 of the main text.

Our primary objective was to assess the impact of the intervention on TB care pathways, where ensuring comparability of the underlying population across time periods was key. To that end, our regression models included a range of relevant covariates and yielded statistically significant results. We also conducted robustness checks using quantile regression, which produced consistent findings.

Given this, we believe that post-stratification or response weighting would have added only marginal analytical value and would have been unlikely to meaningfully alter the inference on treatment effects.

- Endline control and intervention groups differ in age, occupation, and comorbidities. This threatens internal validity unless more rigorous covariate balancing is applied.

Thank you for raising this point. While a few covariates—such as age (p = 0.003) and occupation (p = 0.021)—differ statistically between endline control and intervention groups, these differences are modest in magnitude and not unexpected in a real-world quasi-experimental study. Most other characteristics, including gender, household type, and TB history, are balanced across groups. Some differences in patient delay, diagnostic delay, and provider type are expected, as they are part of the causal pathway influenced by the intervention itself.

To address any potential bias from baseline differences, we included all key demographic and clinical covariates in our regression models. These adjustments ensure that the estimated intervention effect is not confounded by observed differences. In addition, we conducted robustness checks using quantile regression, which yielded consistent and stable results.

## 8. Clustering Effects:

- Patients are nested within providers and likely within communities. No adjustments for clustering are made in regression models. This may underestimate standard errors.

We agree that patients are likely clustered by provider and community, and that such clustering can influence standard error estimates. However, while we have not made formal adjustments for clustering due to the absence of consistent provider or community identifiers across baseline and endline datasets, the type of provider first consulted (e.g., allopathic, AYUSH, informal, pharmacy) is included as a covariate in the model. This partially accounts for between-provider variation.

In addition, we used robust (heteroskedasticity-consistent) standard errors, which help mitigate variance misspecification, and conducted quantile regression. The latter is less sensitive to heteroskedasticity and support the consistency of our findings.

9. Qualitative Component:
• The qualitative data are rich but under-integrated with quantitative findings. More structured triangulation (e.g., using joint display tables) would improve interpretation.

Thank you for this helpful suggestion.

In our submission, qualitative data were thematically coded and used to construct key quantitative variables, including *first point of contact, reasons for provider choice, and financial burden*. These were synthesized from multiple interview responses and cross-validated during transcription to ensure internal consistency.

We had also included Figure 1 which collates the overall patient pathway observed through the intervention. In this version, however – we have revised the same to be simpler and more interpretable, while also including an additional joint table, supporting the triangulation of findings.

The discussion section has also been made more concise, in line of the above additions / changes. .

We hope these revisions improve integration and interpretation of the qualitative insights.

• The findings could be better used to explain why patient delay improved in both control and intervention arms.

As noted in the manuscript, the observed reduction in patient delay across both arms was not expected, given the provider-focused nature of the intervention. Our qualitative and quantitative data did not reveal specific insights to explain this trend conclusively.

However, we hypothesize that broader shifts in care-seeking behaviour post-COVID19, such as increased public awareness or improved health system responsiveness, may have contributed. In the absence of definitive evidence, we have interpreted this as a potential reflection of wider systemic or behavioural changes not directly captured in our study.

10. Others
• What proportion of total presumptive TB patients referred by informal providers eventually received a TB diagnosis?

Eight percent of total presumptive TB patients referred by informal providers eventually received a TB diagnosis.

• Were there dropouts among the informal or AYUSH providers during the project? If so, what were the reasons and how were they handled?

Yes, there were dropouts – and it is typical for any private sector / practitioner engagement program.

For AYUSH, this included concerns over the limited integration of traditional systems in mainstream TB protocols. For informal practitioners – a major concern was the fear of legal repercussions or scrutiny due to their unofficial status.

Dropouts were observed even for allopathic practitioners. The reason, broadly, was the non-sufficiency of the financial and non-financial (administrative) support provided by the project team.

For instance, for high burden practitioners – the added exercise of attending CMEs or reporting requirements – was a disincentive, and the financial incentives were not commensurate with the same.

The continuous engagement of providers was one of the core-activities for the project team – in order to minimize, or reverse any observed attrition. One strategy utilized was the advocation of AYUSH and unqualified providers as key informants for the NTEP program, which motivated their continued engagement. Additionally, actively engaged practitioners were often called upon as local champions in meetings, in order to improve peer motivation and retention. This included distribution of certificates – and community level recognition.

Other activities to ensure engagement were regular visits by field staff, and adapting CMEs based on providers’ suggestions and their practice contexts.

Clarification on Ethical Review Committees: The manuscript states that ethical approvals were obtained from two different Institutional Ethics Committees for the baseline and endline assessments. For the baseline phase, ethics approval was obtained from Sigma IRB the endline phase, ethics approval was obtained from TRIOs IRB .The rationale for using two different IRBs should be clarified. It would also be helpful to confirm whether there were any protocol deviations, differences in consent forms, or interviewer procedures between the two studies due to differing IRB requirements.

**Thank you for your comment.** The two Institutional Review Boards (IRBs) were engaged separately because the baseline and endline assessments were conducted two years apart, and each phase was implemented by a different research agency selected through an open Request for Proposals (RFP) process institutionalized by the William J. Clinton Foundation (WJCF). At the time of baseline, the implementing partner obtained ethical approval from Sigma IRB; for the endline, a different agency secured approval from TRIOs IRB.

There were no protocol deviations between the two phases. However, the endline questionnaire included a few additional questions to capture changes in program context and implementation experience. Consent procedures and interviewer protocols remained consistent and adhered to standard ethical guidelines.

Reviewers' comments:

Reviewer's Responses to Questions

**Comments to the Author**

1. Does this manuscript meet PLOS Global Public Health’s [publication criteria](https://journals.plos.org/globalpublichealth/s/criteria-for-publication)? Is the manuscript technically sound, and do the data support the conclusions? The manuscript must describe methodologically and ethically rigorous research with conclusions that are appropriately drawn based on the data presented.

Reviewer #1: Yes

Thank You

2. Has the statistical analysis been performed appropriately and rigorously?

Reviewer #1: Yes

Thank You

3. Have the authors made all data underlying the findings in their manuscript fully available (please refer to the Data Availability Statement at the start of the manuscript PDF file)?

The [PLOS Data policy](https://journals.plos.org/globalpublichealth/s/data-availability) requires authors to make all data underlying the findings described in their manuscript fully available without restriction, with rare exception. The data should be provided as part of the manuscript or its supporting information, or deposited to a public repository. For example, in addition to summary statistics, the data points behind means, medians and variance measures should be available. If there are restrictions on publicly sharing data—e.g. participant privacy or use of data from a third party—those must be specified.

Reviewer #1: No

Dear Reviewer, We have indeed, provided the data on a GitHub website, at this link: <https://github.com/ridhimasodhi/PatientPathway>

We are in alignment that underlying data, for the best extent possible, should be provided in a public repository, for the benefit of readers, reviewers and students as well.

4. Is the manuscript presented in an intelligible fashion and written in standard English?

PLOS Global Public Health does not copyedit accepted manuscripts, so the language in submitted articles must be clear, correct, and unambiguous. Any typographical or grammatical errors should be corrected at revision, so please note any specific errors here.

Reviewer #1: Yes

Thank You

5. Review Comments to the Author

Please use the space provided to explain your answers to the questions above. You may also include additional comments for the author, including concerns about dual publication, research ethics, or publication ethics. (Please upload your review as an attachment if it exceeds 20,000 characters)

Reviewer #1: Well presented manuscript, the methodology was good and the analysis precise and clear. Data availability statement has not been added.
Is there a reason why weighting or sensitivity analysis was not done to control for high non-response rate?

Thank You for your question. As we have shared above, we have indeed provided the data (GitHub).

On the high non-response rate => We acknowledge the high non-response rate (~70%). However, we employed stratified random sampling based on age, gender, and provider type to maintain the representativeness of the underlying population. This is reflected in Table 1 of the main text.

Our primary objective was to assess the impact of the intervention on TB care pathways, where ensuring comparability of the underlying population across time periods was key. To that end, our regression models included a range of relevant covariates and yielded statistically significant results. We also conducted robustness checks using quantile regression, which produced consistent findings.

Given this, we believe that post-stratification or response weighting would have added only marginal analytical value and would have been unlikely to meaningfully alter the inference on treatment effects.

6. PLOS authors have the option to publish the peer review history of their article ([what does this mean?](https://journals.plos.org/globalpublichealth/s/editorial-and-peer-review-process#loc-peer-review-history)). If published, this will include your full peer review and any attached files.


**Do you want your identity to be public for this peer review?** If you choose “no”, your identity will remain anonymous but your review may still be made public.


For information about this choice, including consent withdrawal, please see our [Privacy Policy](https://www.plos.org/privacy-policy).

Reviewer #1: No
