# Supplementary material for: Strengthening TB care pathways: Integrating informal and AYUSH practitioners for early diagnosis and referral
Source: PLOS Glob Public Health. 2026 Apr 8;6(4):e0004416. doi: 10.1371/journal.pgph.0004416 (PMC13061243; doi:10.1371/journal.pgph.0004416)
Supplement: Attachment — Submitted filename: Response_to_Reviewers_auresp_3.docx [file pgph.0004416.s005.docx]

Third Revision

19th January 2026

**Note of thanks**

We sincerely thank the reviewers for taking the time to read through our manuscript, and giving such detailed feedback. We have revised the manuscript basis the latest comments, and have also included an additional sub-section on future research.

Additional Editor Comments (if provided):

***Authors’ responses to editor’s and reviewer’s comments are mentioned in blue***

[Note: HTML markup is below. Please do not edit.]

Reviewers' comments:

*Reviewer's Responses to Questions*

**Comments to the Author**

1. If the authors have adequately addressed your comments raised in a previous round of review and you feel that this manuscript is now acceptable for publication, you may indicate that here to bypass the “Comments to the Author” section, enter your conflict of interest statement in the “Confidential to Editor” section, and submit your "Accept" recommendation.

Reviewer #4: (No Response)

Reviewer #5: (No Response)

2. Does this manuscript meet PLOS Global Public Health’s [publication criteria](https://journals.plos.org/globalpublichealth/s/criteria-for-publication)? Is the manuscript technically sound, and do the data support the conclusions? The manuscript must describe methodologically and ethically rigorous research with conclusions that are appropriately drawn based on the data presented.

Reviewer #4: Yes

Reviewer #5: Yes

3. Has the statistical analysis been performed appropriately and rigorously?

Reviewer #4: Yes

Reviewer #5: Yes

4. Have the authors made all data underlying the findings in their manuscript fully available (please refer to the Data Availability Statement at the start of the manuscript PDF file)?

The [PLOS Data policy](https://journals.plos.org/globalpublichealth/s/data-availability) requires authors to make all data underlying the findings described in their manuscript fully available without restriction, with rare exception. The data should be provided as part of the manuscript or its supporting information, or deposited to a public repository. For example, in addition to summary statistics, the data points behind means, medians and variance measures should be available. If there are restrictions on publicly sharing data—e.g. participant privacy or use of data from a third party—those must be specified.

Reviewer #4: Yes

Reviewer #5: Yes

5. Is the manuscript presented in an intelligible fashion and written in standard English?

PLOS Global Public Health does not copyedit accepted manuscripts, so the language in submitted articles must be clear, correct, and unambiguous. Any typographical or grammatical errors should be corrected at revision, so please note any specific errors here.

Reviewer #4: Yes

Reviewer #5: Yes

6. Review Comments to the Author

Please use the space provided to explain your answers to the questions above. You may also include additional comments for the author, including concerns about dual publication, research ethics, or publication ethics. (Please upload your review as an attachment if it exceeds 20,000 characters)

Reviewer #4: A good study showing how integration of informal and AYUSH practitioners helps in early diagnosis and referral of tuberculosis. Similar approach was practiced in other diseases like kala-azar as well. Having said this, a word of caution is also required to limit informal and AYUSH practitioners to referral only, and not towards treatment modalities other than ATT. During implementation, local practitioners should be strictly advised against indiscriminate use of antibiotics, steroids or other AYUSH treatments before or after diagnosis.

Reviewer #5: I am reviewing Revision 2. I have not seen the previous versions of this manuscript, nor have I submitted any previous suggested revisions. I do not have access to the reviewer comments from previous reviewers, but there was clearly a significant revision in the manuscript, based in previous reviewers comments.

In addition, Revision 2 does not have manuscript line numbering, so I can only refer to page numbers for suggested changes.

This manuscript describes a "quasi-experimental" study that evaluates the impact of an intervention that links non-allopathic health providers, including traditional healers and AYUSH practitioners, with the formal allopathic medical community to improve the diagnosis and treatment of pulmonary tuberculosis. The final conclusions highlight that there was a significant reduction in the delay in diagnosis of TB due to the intervention.

GENERAL COMMENTS

1. The authors do describe the intervention with non-formal traditional providers but it would be helpful to have much more detail. How long did this intervention take? (weeks, months??) How much did it cost? How were the various target audiences of non-allopathic "healers" identified and who provided the information about referrals, access to CXR, etc? Was feedback provided to these traditional healers after the study was completed? Did the non-allopathic healers feel positive about this interaction and did they have any useful suggestions for future action? What would the authors have done differently? This would be helpful for readers who are contemplating a similar exercise in their own setting.

Thanks for these question. Project ADITYA was implemented under the Wave 8 of the TB Reach initiative ([Link](NULL)) and was conducted between January 2021 and September 2022 (~1.5 years).

While the total grant budget for the implementing agency was USD 400,000 (reported in the Funding section), this covered multiple intervention components. We have, hence, not discussed or spoken about costs – as any such discussion would also require disaggregation, which is not feasible with data made available for this particular study.

On your point on the engagement with providers, especially with non-allopathic providers, multiple questions around this subject (e.g., how were referrals made, how were they validated, how many referrals by which type of provider) were answered during an earlier review round. These details, however, were not provided in detail in the manuscript, as that would require dwelling into multiple operational aspects of the program, which is outside the focus of this particular study.

However, based on your comments, we have further edited the “*Drug Sensitive TB Care services under Project Aditya*” section under Methods, and it now has additional details on the intervention.

We did not collect any formal feedback on the experience of non-allopathic providers, and hence providing that information would not be feasible. Having said that, programmatic experience suggests that majority viewed this interaction positively, particularly the opportunity to participate in CME activities and referral networks. This comment has prompted us to include a sub-section on “future research”, which is now placed in the revised manuscript.

Thank You!

SPECIFIC COMMENTS:

1. Pages 1-2. I would suggest removing the AUTHOR SUMMARY. It is duplicative of the Abstract and the Introduction.

Thanks – we agree, but an author summary is a requirement by the journal.

2. Page 2, INTRODUCTION: Fourth sentence, replace, "...well-known..." with "...common..."

Thanks, done.

3. Page 2 INTRODUCTION-8th sentence, it's not clear what the phrase, "...the same continues to...."means. Can you rewrite this?

Thank You for this feedback – the line does seem to lack clarity.

We have rephrased this slightly --- replaced “the same” with “these factors”. This makes it more clear that we are talking about the multitude of factors causing diagnostic delay.

“Provider shopping, and misdiagnosis, from unqualified as well as qualified providers, is common, and often results in prolonged delays, stretching for weeks or months before treatment begins [8–11]. Compounded further by non-specific antibiotic use and delay in diagnostic testing, **these factors continue** to be detrimental to TB elimination efforts within and outside India [8,12–18]. While significant provider-engagement efforts have been made [19–22], not many have integrated non-allopathic practitioners [2,4]”

4. Page 2 INTRODUCTION, Line 10, add "where" after the word "region."

Thank You – this is very detailed feedback, and we are grateful. Edited.

5. Page 2, INTRODUCTION, Line 11, can you state what this proportion of 4.4% and 1.4% refers to. Is this of the Total population of health seeking persons?

Thank you – this refers to the total population who were treated for an ailment; and comes from the 75^th^ round of National Statistical Office; and the latest comprehensive information on social consumption. We have revised the statement to be more clear as below:

*“In Chhattisgarh, the region where this study is based, the breakdown of treated ailments by type of healthcare provider indicates that reliance on AYUSH (Ayurveda, Yoga & Naturopathy, Unani, Siddha, and Homeopathy) practitioners is 4.4% in rural areas and 1.4% in urban areas”*

6. Page 3, METHODS, What is the meaning of "DS" in the subtitle?

DS stands for Drug Sensitive – as our study is based on patients seeking treatment for Drug Sensitive Tuberculosis. Thanks for pointing this out, as it should have had the full form (now edited).

7. Page 8, RESULTS, Demographic survey,. The authors state that the majority of patients in this study are male. Would they comment on possible differences if there were more female subjects involved?

Thanks for this – the observed gender distribution in our study sample is actually is consistent with the male predominance among notified TB patients in India and is therefore broadly representative.

Gender (as well as age) is actually included as a covariate in the three regression models – to assess if they meaningfully impact the outcomes of interest. Neither of these were found significant. This is expected as other factors like occupation, symptoms and their severity, as well as comorbidities, are all also influenced by age. Under these considerations, we have not commented on possible differences due to age or gender.

8. Page 16 TABLE 6. This was a very useful and interesting table. Congratulations

Thank You!

9. Page 17, under "PATIENT DELAY......." Line 3 should be revised to read "......visit a medical professional based on (or not) advice given by close contacts....."

Thank You, we have edited this.

7. PLOS authors have the option to publish the peer review history of their article ([what does this mean?](https://journals.plos.org/globalpublichealth/s/editorial-and-peer-review-process#loc-peer-review-history)). If published, this will include your full peer review and any attached files.


**Do you want your identity to be public for this peer review?** If you choose “no”, your identity will remain anonymous but your review may still be made public.


For information about this choice, including consent withdrawal, please see our [Privacy Policy](https://www.plos.org/privacy-policy).

Reviewer #4: **Yes:** Dr. Madhuri Devaraju

Reviewer #5: **Yes:** Paul R De Lay, MD, DTM&H (Lond)

[NOTE: If reviewer comments were submitted as an attachment file, they will be attached to this email and accessible via the submission site. Please log into your account, locate the manuscript record, and check for the action link "View Attachments". If this link does not appear, there are no attachment files.]

Figure Resubmissions:

While revising your submission, we strongly recommend that you use PLOS’s NAAS tool (https://ngplosjournals.pagemajik.ai/artanalysis) to test your figure files. NAAS can convert your figure files to the TIFF file type and meet basic requirements (such as print size, resolution), or provide you with a report on issues that do not meet our requirements and that NAAS cannot fix.

Our image files are already revised using PLOS’s earlier tool – but we have now re-edited them using this tool.

After uploading your figures to PLOS’s NAAS tool - https://ngplosjournals.pagemajik.ai/artanalysis, NAAS will process the files provided and display the results in the "Uploaded Files" section of the page as the processing is complete. If the uploaded figures meet our requirements (or NAAS is able to fix the files to meet our requirements), the figure will be marked as "fixed" above. If NAAS is unable to fix the files, a red "failed" label will appear above. When NAAS has confirmed that the figure files meet our requirements, please download the file via the download option, and include these NAAS processed figure files when submitting your revised manuscript.
